# Telomere-to-telomere genome assembly of *Talaromyces purpureogenus* strain ISA502 isolated from maize rhizosphere

**DOI:** 10.1128/mra.00122-25

**Published:** 2025-06-18

**Authors:** Jun Zhou, Maria Elena Antinori, Gabriele Bellotti, Michel Chalot, Edoardo Puglisi

**Affiliations:** 1 Université Marie et Louis Pasteur, CNRS, Chrono-environnement (UMR 6249), Montbéliard, France; 2 Department for Sustainable Food Process, Università Cattolica del Sacro Cuore, Piacenza, Italy; 3 ISA Innovations for Sustainable Agriculture R&D, Piacenza, Italy; University of California Riverside, Riverside, California, USA

**Keywords:** *Talaromyces purpureogenus*, genome, maize

## Abstract

We present a telomere-to-telomere genome assembly of *Talaromyces purpureogenus* strain ISA502, isolated from the maize rhizosphere. The 30.33 Mb genome, assembled using hybrid sequencing, comprises 8 chromosomes with 99% BUSCO completeness, offering improved resolution over existing fragmented genomes and advancing studies on its ecological and biotechnological significance.

## ANNOUNCEMENT

The rhizosphere hosts diverse fungi essential to nutrient cycling and plant health. Among these, *Talaromyces purpureogenus* stands out for its ability to produce bioactive metabolites such as antimicrobial compounds, pigments, and enzymes, making it highly relevant for agricultural and biotechnological applications (1, 2). Although six genome sequences of *T. purpureogenus* are publicly available, they remain fragmented (3), limiting structural resolution and downstream analyses. Here, we report the chromosome-scale genome assembly of *T. purpureogenus* strain ISA502, representing a significant improvement over existing draft genomes.

**Fig 1:**
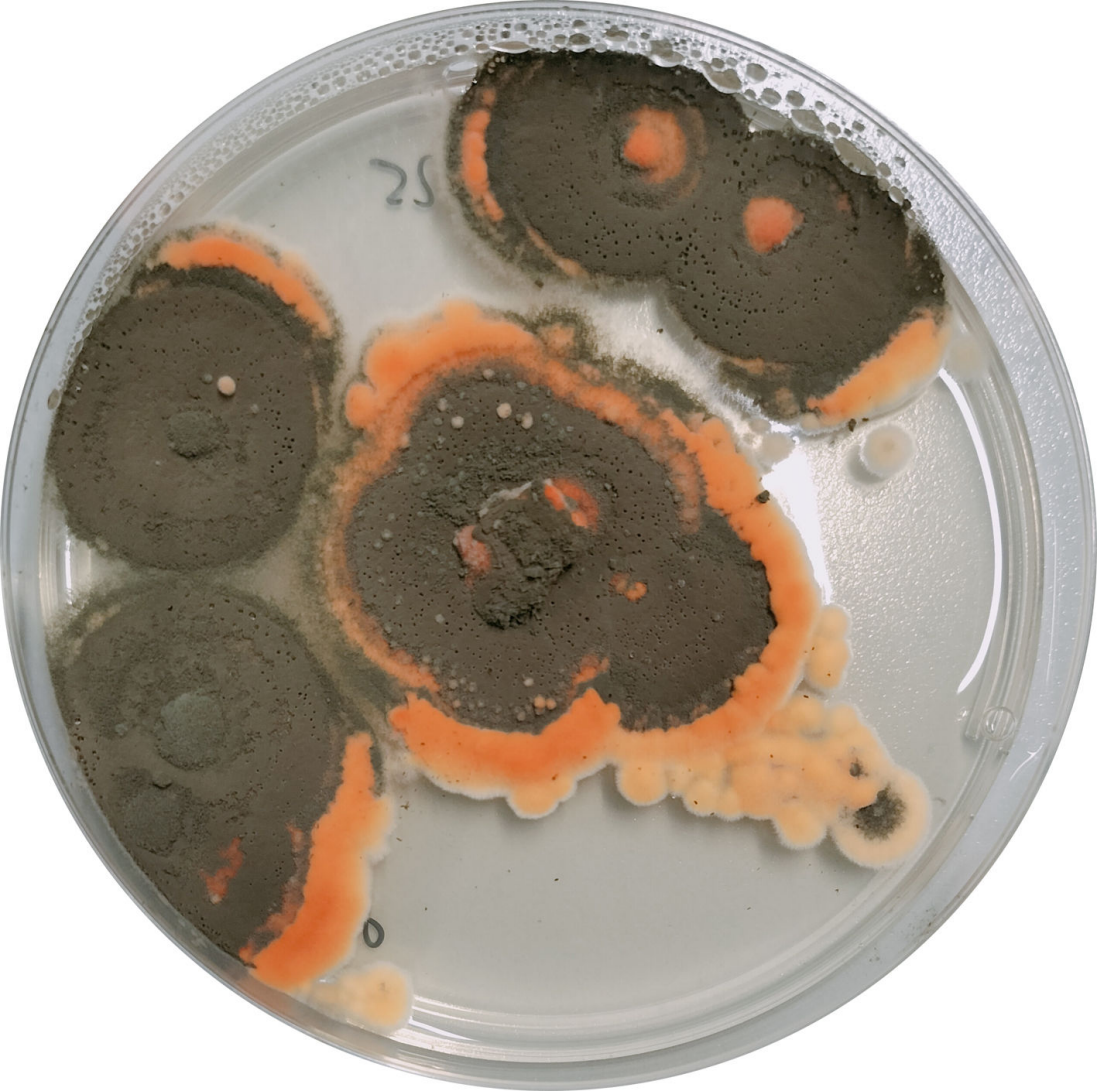
*Talaromyces purpureogenus* ISA502 grown 10 days on MEA (upper side of the plate). From the bottom, the strain appears pale yellow.

Strain ISA502 was isolated from the maize rhizosphere in Pavia, Italy (GPS: N 45.137293, E 8.776014) following the protocol of Barillot et al. (4). Briefly, rhizospheric soil was suspended in 0.9% NaCl solution, and dilutions of the suspension were plated on malt extract agar (MEA). Single mycelial colonies were subcultured on MEA, and strain purity was confirmed via single-spore isolation. Mycelial cultures were grown on MEA at 28°C (Fig. 1), and genomic DNA was extracted using the HiPure Universal DNA Kit D301 (Genepioneer Biotechnologies). A hybrid sequencing strategy was employed, combining Illumina short reads for base-level accuracy and Oxford Nanopore Technologies (ONT) long reads for resolving complex genomic structures. DNA libraries for Illumina sequencing were prepared using the VAHTS Universal DNA Library Prep Kit for Illumina V3 ND607 (Vazyme). Sequencing was performed using the Illumina NovaSeq 6000 platform (PE150), yielding 19,028,384 reads and 5.71 Gb of data with an average depth of coverage of ~177×. Quality control, performed with fastp (v0.20.0) (5), removed adapter sequences, low-quality reads (Q5), and reads with over five ambiguous bases. For ONT sequencing, genomic DNA was purified using 0.4× magnetic beads to remove short fragments (<2 kb). Libraries were prepared with the SQK-LSK110 ligation kit and sequenced on a PromethION 48 system with R9.4.3 flow cells. The run yielded 419,591 reads totaling 4.95 Gb with a read N50 of 21,477 bp (~152× depth). Reads were basecalled with Guppy (hac) and filtered using Filtlong (v0.2.1) to remove low-quality or <1,000 bp reads. Assembly began with self-correction of ONT reads using Canu (v2.1.1) (6), followed by hybrid error correction using LoRDEC (v0.9) (7) with Illumina reads. Corrected long reads were assembled using NextDenovo (v2.5) (8). Misassemblies were identified and resolved manually by aligning ONT reads with minimap2 (v2.1) (9) and inspecting dot plots generated by MUMmer4 to assess synteny and structural inconsistencies. The genome was polished in three rounds using NextPolish (v1.4.0) (10) with ONT reads, and then in three rounds using Pilon (v1.23) (11) with Illumina reads. Default parameters were used for all software unless otherwise specified.

The final assembly consists of 8 chromosomes totaling 30.33 Mb, with a GC content of 44.44% and an N50 of 4.12 Mb. The largest chromosome spans 5.80 Mb. Assembly completeness, evaluated using BUSCO (v5.2.1) (12), showed 99% completeness (97.9% single copy, 1.1% duplicated, 0.1% fragmented, and 0.9% missing) (Table 1). Read coverage analysis using minimap2 showed uniform coverage across the genome. Telomeric repeats (CCCTAA)n were detected at both ends of all contigs, indicating complete chromosomal assemblies.

**TABLE 1:** Genome sequencing statistics

Parameter	Value
Illumina sequencing	
Average depth of coverage, Illumina short reads	~177×
Reads number	19,028,384
Total bases (bp)	5,708,515,200
Nanopore sequencing	
Average depth of coverage, ONT long reads	~152×
Reads number	419,591
Total bases (bp)	4,950,531,796
N50 read length (bp)	21,477
Genome assembly	
No. of chromosomes	8
Total length (bp)	30,326,944
Largest chromosome	5,800,888
GC (%)	44.44
N50 (bp)	4,121,854
L50	4
Genome assembly assessment	
Complete BUSCOs	99%
Complete and single-copy BUSCOs	97.90%
Complete and duplicated BUSCOs	1.10%
Fragmented BUSCOs	0.10%
Missing BUSCOs	0.90%

## Data Availability

The genome and sequencing data have been deposited at NCBI under BioProject accession PRJNA1215053. The BioSample accession is SAMN46395145. The Illumina sequencing reads are available in the NCBI Sequence Read Archive under accession SRR32327772, while the nanopore sequencing reads are accessible under accession SRR32327919. This Whole Genome Shotgun project has been deposited in DDBJ/ENA/GenBank under the accession no. JBLFET000000000. The version described in this paper is the first version, JBLFET010000000.
